# Neural dynamics and information representation in microcircuits of motor cortex

**DOI:** 10.3389/fncir.2013.00085

**Published:** 2013-05-03

**Authors:** Yasuhiro Tsubo, Yoshikazu Isomura, Tomoki Fukai

**Affiliations:** ^1^Laboratory for Neural Circuit Theory, RIKEN Brain Science InstituteWako, Saitama, Japan; ^2^Brain Science Institute, Tamagawa UniversityMachida, Tokyo, Japan; ^3^Core Research for Evolutional Science and Technology, Japan Science and Technology Agency, SanbanchoChiyoda-ku, Tokyo, Japan

**Keywords:** synchronization, gamma oscillation, juxtacellular, multiunit, neural code, irregular firing, cortical layer, local circuit

## Abstract

The brain has to analyze and respond to external events that can change rapidly from time to time, suggesting that information processing by the brain may be essentially dynamic rather than static. The dynamical features of neural computation are of significant importance in motor cortex that governs the process of movement generation and learning. In this paper, we discuss these features based primarily on our recent findings on neural dynamics and information coding in the microcircuit of rat motor cortex. In fact, cortical neurons show a variety of dynamical behavior from rhythmic activity in various frequency bands to highly irregular spike firing. Of particular interest are the similarity and dissimilarity of the neuronal response properties in different layers of motor cortex. By conducting electrophysiological recordings in slice preparation, we report the phase response curves (PRCs) of neurons in different cortical layers to demonstrate their layer-dependent synchronization properties. We then study how motor cortex recruits task-related neurons in different layers for voluntary arm movements by simultaneous juxtacellular and multiunit recordings from behaving rats. The results suggest an interesting difference in the spectrum of functional activity between the superficial and deep layers. Furthermore, the task-related activities recorded from various layers exhibited power law distributions of inter-spike intervals (ISIs), in contrast to a general belief that ISIs obey Poisson or Gamma distributions in cortical neurons. We present a theoretical argument that this power law of *in vivo* neurons may represent the maximization of the entropy of firing rate with limited energy consumption of spike generation. Though further studies are required to fully clarify the functional implications of this coding principle, it may shed new light on information representations by neurons and circuits in motor cortex.

Neocortical microcircuits have a stereotyped structure, comprising a six-layered network of excitatory pyramidal neurons and inhibitory interneurons. This structure is preserved across many neocortical regions (an exception is agranular areas lacking layer 4: Shepherd, 2009), and is often considered to represent the functional module of cortical information processing. Uncovering how neurons in the different layers process information is a key to understand the principles of cortical computations. Several recent studies have begun to uncover how the dynamics of neural populations underlie motor behavior (Churchland and Shenoy, 2007; Hatsopoulos and Suminski, 2011; Churchland et al., 2012). Unlike the classical view of direction-tuned neurons during reaching (Georgopoulos et al., 1986), direction tuning curves scale with the velocity of arm movement only in a minority of these neurons in primate motor cortex (Churchland and Shenoy, 2007; Hatsopoulos and Suminski, 2011). The results suggest that the relationship between the activity of motor cortex neurons and motor behavior is more divergent and heterogeneous than previously thought. Few studies, however, have evaluated the role of the layered structure of motor cortex in such dynamics. In this paper, we will review the results of our *in vitro* and *in vivo* recording studies that attempt to clarify the characteristic features of neuronal dynamics and information processing in different layers of rat motor cortex.

The first part is devoted to slice recordings of the phase response curves (PRCs) from motor cortex neurons in different layers. The PRC shows the responses of single neurons to a perturbative input and provides a useful mathematical tool for characterizing synchronization properties of a weakly coupled network of arbitrary oscillators (Reyes and Fetz, 1993a,b; Ermentrout, 1996; Ermentrout et al., 2001; Gutkin et al., 2005; Netoff et al., 2005b; Goldberg et al., 2007). Various experimental studies have shown that the local field potential (LFP) or unit activity of the primary motor cortex exhibits gamma-band (30–80 Hz) oscillations during behavior (Sanes and Donoghue, 1993; Murthy and Fetz, 1996; Donoghue et al., 1998; Farmer, 1998). Though these results are mainly from the primate, we also found strong gamma oscillatory components of the LFP and neuronal firing in the motor cortex of behaving rats (Igarashi et al., unpublished observation). Therefore, it is of particular interest to clarify whether the tendency of synchronized oscillatory firing is layer-dependent in motor cortex. Our electrophysiological recordings from a slice preparation of rat motor cortex revealed that the preference to synchronization is layer- and frequency-dependent in rat motor cortex.

In the second part, we explore the relationship between neuronal activity in different cortical layers and motor behavior by conducting simultaneous multiunit recordings and juxtacellular recordings from the motor cortex of rats performing spontaneous voluntary movement (Isomura et al., 2009). The recordings were made in the forelimb area of the rat motor cortex (Donoghue and Wise, 1982; Rouiller et al., 1993; Brecht et al., 2004), where layer 2/3 pyramidal cells principally project to other cortical areas, layer 5 pyramidal cells to subcortical structures such as the spinal cord and the striatum, and layer 6 pyramidal cells to thalamic nuclei. These excitatory pyramidal cells, along with the star-like pyramidal cells in layer 4 of this area, also innervate local cortico-spinal pyramidal cells in layer 5 via axon collaterals (Cho et al., 2004a,b). An *in vitro* study of excitatory laminar connectivity predicted that motor information would flow primarily from layer 2/3 to layer 5 in the primary motor cortex (Weiler et al., 2008). However, the flow of information in the microcircuit of motor cortex requires clarification by recordings from behaving animals. The role of inhibition in motor cortex also needs to be tested by experiment. For instance, the traditional view suggests that inhibitory interneurons inhibit excitatory neurons encoding antagonistic movements (i.e., lateral inhibition). We addressed these issues by combining juxtacellular and multiunit recordings, where the former technique provides accurate spike events and morphological features for recorded neurons (Pinault, 1996; Klausberger et al., 2003; Lee et al., 2004; Mallet et al., 2006; de Kock et al., 2007) and the latter technique enables a simultaneous access to spike events of many neurons (Harris et al., 2000; Isomura et al., 2006; Merchant et al., 2008). Our approach uncovered the functional diversity of pyramidal cells and functional uniformity of fast-spiking (FS) interneurons across all cortical layers in the expression of voluntary movement.

In the third part, we interpret information coding by highly irregular firing of a single motor cortex neuron in terms of a variational principle. While the population signal such as LFP often exhibits oscillatory behavior in the motor cortex (and other cortical areas), activity of single neurons is generally highly irregular in various cortical areas including the primary motor cortex. Why local cortical circuits simultaneously exhibit rhythmic oscillatory activity and stochastic irregular firing remains unclear. However, findings on the biological machinery for irregular firing, namely a balanced excitatory and inhibitory synaptic input (Destexhe et al., 2003; Shu et al., 2003), suggest that the stochastic nature of local cortical circuits is essential for information processing by the brain (Rao et al., 2002; Ma et al., 2006; Berkes et al., 2011; Buesing et al., 2011; Teramae et al., 2012). We closely inspect the statistical properties of the irregular spike generation by cortical neurons. Based on our observations, we propose the constrained maximization of firing-rate entropy (CMFE) as a hypothesis for the neural code.

## Phase response curves: motor cortex neurons

Neurons firing periodically can be regarded as an oscillator. The PRC describes the response of an oscillator to a weak perturbation given at a certain phase of oscillation θ = 2π*t*/*T*, where *t* and *T* denote the time from the previous spike and the period of repetitive firing, respectively (Kuramoto, 1984; Smeal et al., 2010). Depending on the stimulus time, the subsequent spike will be generated at an earlier or a later time. If a small perturbed current injected to a neuron advances the time of spike firing in the subsequent oscillatory cycle, the corresponding PRC takes a positive value at the phase angle. If the current delays the next spike, the value of PRC is negative. The PRC of a neuron can be classified into type 1 or type 2 (Hansel et al., 1995), depending on whether the curve almost always takes positive values (type 1) or takes both positive and negative values (type 2).

The PRC is of interest from a viewpoint of the network dynamics since it has crucial information about rhythmic synchronization (Kuramoto, 1984; Hansel et al., 1995; Ermentrout, 1996; Ermentrout et al., 2001; Nomura et al., 2003; Galan et al., 2005; Gutkin et al., 2005; Netoff et al., 2005a,b; Takekawa et al., 2007; Tsubo et al., 2007b), and several methods have been proposed for computing the PRC from model and experimental data (Torben-Nielsen et al., 2010). In general, neurons with a type-2 PRC can easily be synchronized when they are mutually coupled via fast excitatory synaptic connections, while those with a type-1 PRC may not perfectly be synchronized (Hansel et al., 1995). However, the stable phase difference calculated with the type-1 PRC can be close to zero at low firing rates (Tsubo et al., 2007a). Therefore, the relationship between the PRC types and the synchronization properties is not strict. Experimentally, pyramidal neurons in layer 5 of the cat motor cortex exhibited a type-1 PRC (Reyes and Fetz, 1993a,b), whereas glutamatergic stellate cells in layer 2 of the rat entorhinal cortex (Netoff et al., 2005b), pyramidal neurons in rat hippocampal CA3 (Lengyel et al., 2005) and fast spiking interneurons in somatosensory cortex (Tateno et al., 2007) displayed a predominantly type-2 PRC. In mouse visual cortex, the PRC of pyramidal neurons in layer 2/3 were switched from type 2 to type 1 by cholinergic action (Stiefel et al., 2008). Thus, neuromodulators can change the PRC type.

**Figure 1:**
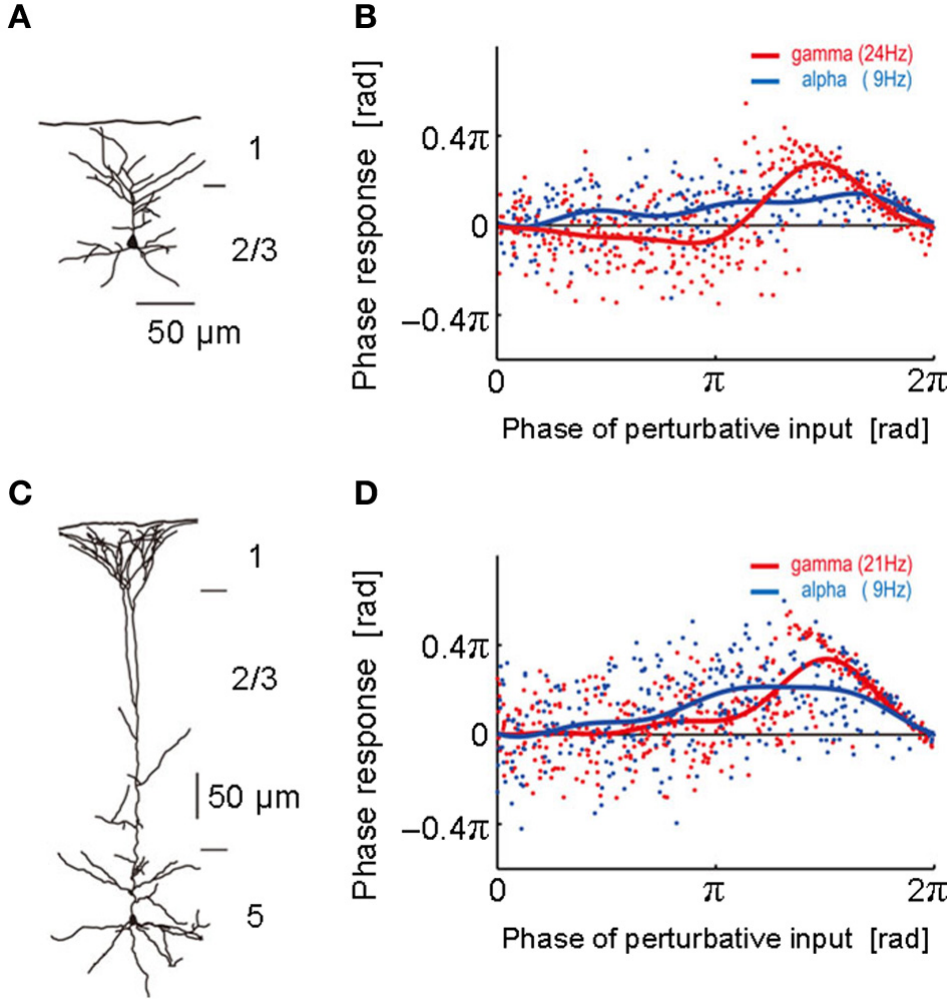
**Phase response curves obtained from different layers. (A)** The morphological structure of a layer 2/3 pyramidal neuron was reconstructed. **(B)** Phase response curves obtained in the alpha (blue; 9 Hz) and gamma (red; 24 Hz) frequency ranges of the neuron described in **(A)**. The abscissa represents the phase at which the neuron was stimulated by a perturbative input and the ordinate the phase response to it. Each dot represents a noisy phase response to a stimulus. Blue and red solid curves display the average PRC obtained by the least-mean-square method. **(C,D)** The morphological structure of a layer 5 pyramidal neuron and its phase response curves obtained in the alpha (blue; 9 Hz) and gamma (red; 21 Hz) frequency ranges. [Modified from Tsubo et al. (2007a)].

**Figure 2:**
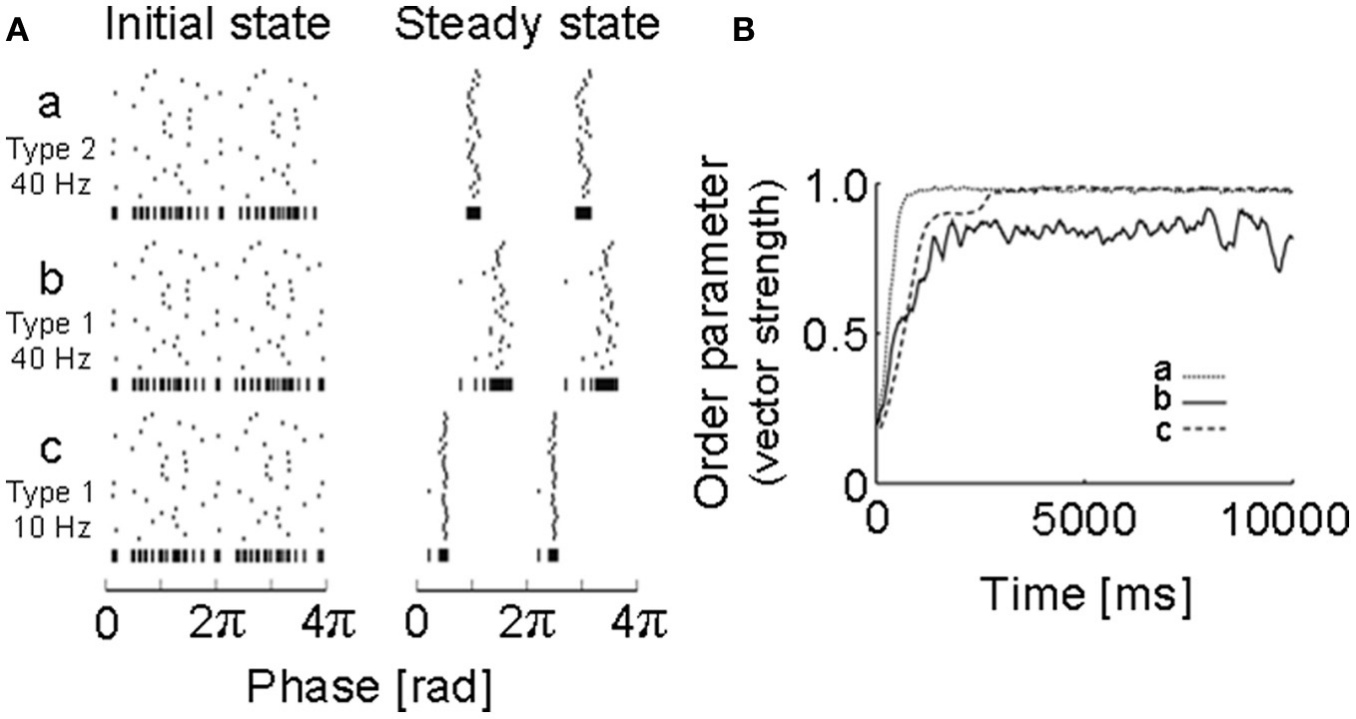
**Simulations of neural oscillators coupled with a PRC obtained for a motor cortex neuron. (A)** Initial states and steady states were obtained by numerical simulations of 30 neuronal oscillators coupled mutually via fast AMPA synapses: a, 40 Hz (type 2); b, 40 Hz (type 1); c, 10 Hz (type 1). Vertical bars at the bottom of each raster plot represent all spikes of the 30 neurons in one sequence to visualize the degree of synchrony. **(B)** How the degree of synchrony evolved in the 30-neuron networks shown in **(A)** was presented using the phase order parameters, which are equal to unity when all oscillators are entrained into perfect in-phase synchronization, while it is zero when they exhibit asynchronous oscillations. [Modified from Tsubo et al. (2007a)].

To obtain the PRC, we performed intracellular and whole-cell patch-clamp recordings at the soma of layer 2/3 and layer 5 pyramidal neurons of the rat motor cortex (Tsubo et al., 2007a) (Figure 1). Although cortical neurons showed a variety of responses to a step current injection, we only examined those neurons that displayed near-periodic firing patterns. We tested the PRC of pyramidal neurons in theta (4–8 Hz), alpha (8–13 Hz), beta (13–20 Hz), and gamma (20–45 Hz) frequency ranges. The PRC depended on the range of firing rates and the cortical layers to which neurons belong. In the theta, alpha and gamma frequency ranges, layer 2/3 or layer 5 pyramidal neurons tend to possess type-2 or type-1 PRCs, respectively. In the beta frequency ranges, the PRCs display type-1 properties in both layer 2/3 and layer 5 pyramidal neurons. However, simulations of coupled oscillators showed that the stable phase difference almost vanishes at such low frequencies even if the PRC belongs to type 1 (Figure 2).

The implications of the above results for oscillatory synchronization are as follows. In the gamma frequency range, which is of particular cognitive importance (Ward, 2003; Herrmann et al., 2004), recurrent AMPA synaptic connections possibly promote synchronous neuronal firing of layer 2/3 pyramidal neurons, but not that of layer 5 pyramidal neurons. Compared to the gamma frequency range, the differences in the PRC type will not be crucial for synchronous firing in the theta, alpha and beta frequency ranges owing to the long time scales of the oscillation period relative to the decay constant of the AMPA receptor-mediated synaptic current.

In summary, the PRCs of the population of layer 5 pyramidal neurons displayed no significantly negative phase in all frequency ranges, suggesting that the PRC type of these neurons is primarily type 1. In contrast, the phase responses recorded from layer 2/3 pyramidal neurons constitutes a heterogeneous mixture of the two types. In the superficial layer, the type 2 is dominant phase response type in the gamma frequency range and seems to be dominant also in the theta frequency range. Both type 1 and type 2 appear equally often in the alpha and beta frequency ranges. In 46% of the layer 2/3 neurons and 30% of the layer 5 neurons, the PRC types were different in the different frequency ranges. Implications of the heterogeneous mixture of the PRC types for the dynamics of neuronal synchronization are found in Tsubo et al. (2007b).

## Motor cortex activity during voluntary movements

As described above, we found that the potential ability of single neurons for synchronization is different between superficial and deep layers of the rat motor cortex *in vitro*. Then, how do the motor cortex neurons work cooperatively in order to execute a certain voluntary movement *in vivo*? For example, it is possible that superficial layer neurons of motor cortex may participate in preparation for the movement while deep layer neurons participate in execution of it. It is also possible that excitatory pyramidal cells of motor cortex may drive the movement while inhibitory interneurons simply suppress it. However, it has been technically difficult to morphologically identify an electrophysiologically recorded neuron, or even to distinguish excitatory (pyramidal cells) and inhibitory (interneurons), in awake animals, especially in those performing an operant behavioral task. To address this issue, we first developed an efficient task-training system in which several rats simultaneously and independently learned a behavioral task (e.g., an operant learning of lever manipulation with forelimb) in a head-fixed condition (Isomura et al., 2009; see also Kimura et al., 2012). This enabled us to test a sufficient number of behaving animals for morphological and electrophysiological experiments. We thus combined the behavioral task system with a juxtacellular recording, which is a unique electrophysiological technique to record spiking activity of a single neuron accurately and stably, and later to visualize its morphological structure (Pinault, 1996).

**Figure 3:**
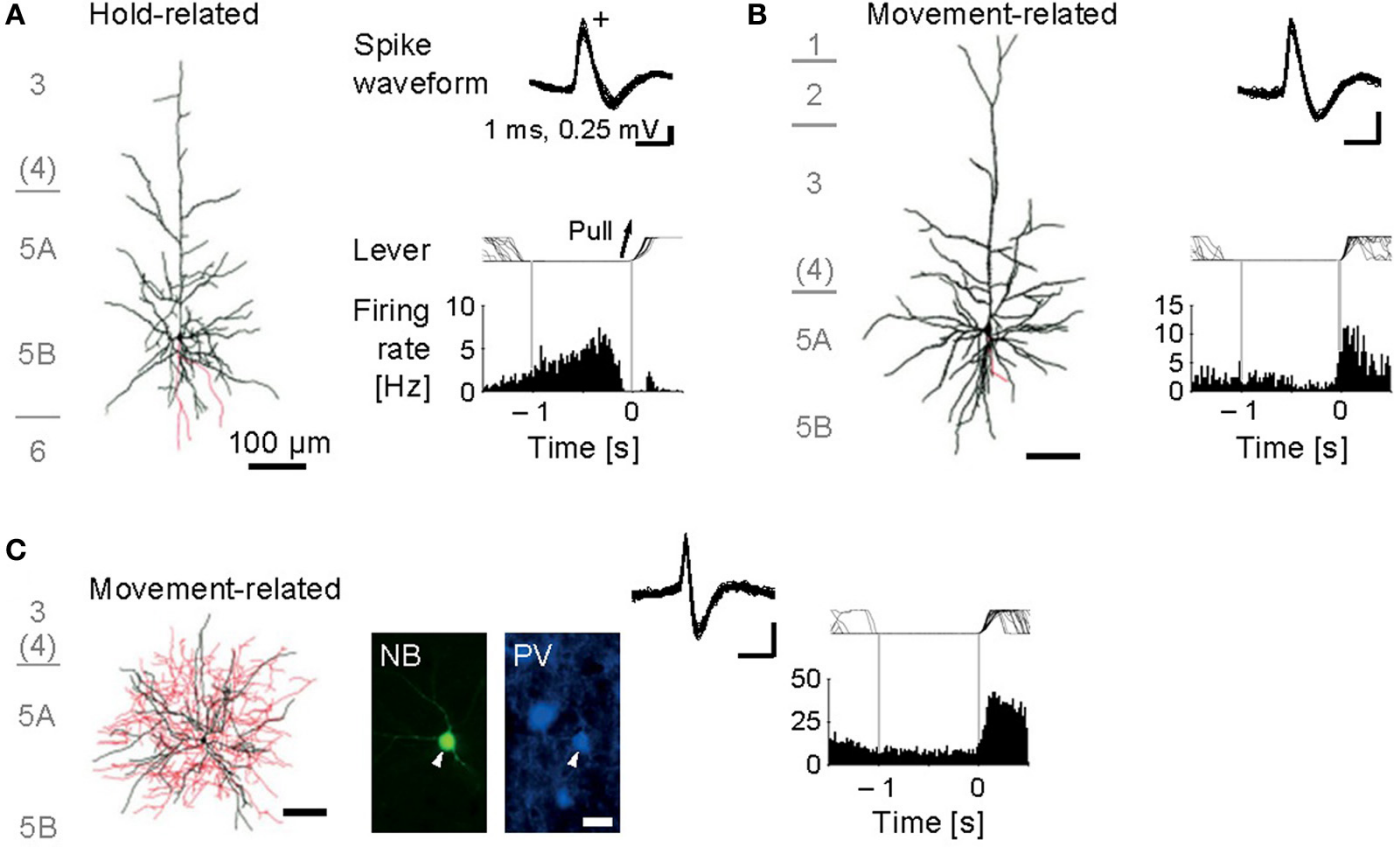
**Functional activity in identified motor cortex neurons. (A)** Juxtacellularly identified layer 5 pyramidal cell that exhibited hold-related activity. Left, visualized soma and dendrites (black) and axons (red). Right, recorded spike waveforms (top) and functional spike activity histogram aligned with the onset of lever pull movement (0 s). **(B)** Layer 5 pyramidal cell with movement-related activity. **(C)** Layer 5 fast-spiking (FS) basket cell with movement-related activity. Bottom, fluorescent images for Neurobiotin (NB) and parvalbumin (PV). [Modified from Isomura et al. (2009)].

Our juxtacellular recordings from the forelimb area of motor cortex during forelimb movements revealed that identified pyramidal cells increased their spiking activity at a variety of timing around the onset of lever pull movement (Isomura et al., 2009). Some of them showed sustained or slowly changing activation in a lever hold period before the movement onset (Figure 3A; namely, hold-related activity), which might be involved in motor preparation or suppression, and others showed phasic activation during the movement (Figure 3B; movement-related activity), which might be involved in motor execution or sensory feedback. Thus, the pyramidal cells of motor cortex participate in several different functions for a voluntary movement. Importantly, we revealed the pyramidal cells with hold-related activity were not restricted to a specific layer, but in all the layers of motor cortex except for layer 1. The pyramidal cells with movement-related activity were also present in layers 2 through 6 of motor cortex. Though recent work in primate motor cortex suggests that relationships between neuronal activity and motor behavior are generally complex (Churchland et al., 2012), the execution and preparation phases of movement likely represent distinct task demands and may be treated separately, as in the present task. It is noteworthy that Weiler et al. (2008) indicated that an excitatory connection from superficial (2/3) layers to deep (5) layer is the strongest pathway between distinct layers of the motor cortex. Thus, the movement-related pyramidal cells in the layer 2/3 may send their motor information to those in the layer 5 to execute the movement.

Now we turn to the functional activity of a major subclass of neocortical GABAergic interneurons, i.e., FS interneurons including the basket cells and chandelier cells morphologically, and often expressing the calcium-binding protein parvalbumin as an FS neuron-specific marker (Markram et al., 2004). We obtained juxtacellular recordings from the FS interneurons of motor cortex during the voluntary movement. In contrast to the functional diversity of pyramidal cells, most of the FS neurons dominantly exhibited the phasic movement-related activity in relation to the voluntary movement (Figure 3C; Isomura et al., 2009). It is therefore unlikely that the FS neurons simply suppress actual muscular movements through inhibitory synaptic transmissions. One may consider two possibilities to account for their phasic activation during voluntary movements. One possibility is “balanced inhibition” (feedforward inhibition), in which inhibitory FS neurons shape a motor command together with excitatory pyramidal cells. This hypothetical function is similar to balanced inhibition observed in the auditory cortex (Wehr and Zador, 2003) and somatosensory cortex (Okun and Lampl, 2008). The other is “recurrent inhibition” (feedback inhibition), in which excitatory pyramidal cells for a specific movement selectively inactivate nearby neurons coding unnecessary movements via collateral activation of inhibitory neurons (Georgopoulos and Stefanis, 2007). In any case, the FS neurons do not extinguish a voluntary movement, but actively elaborate it in cooperation with the pyramidal cells.

**Figure 4:**
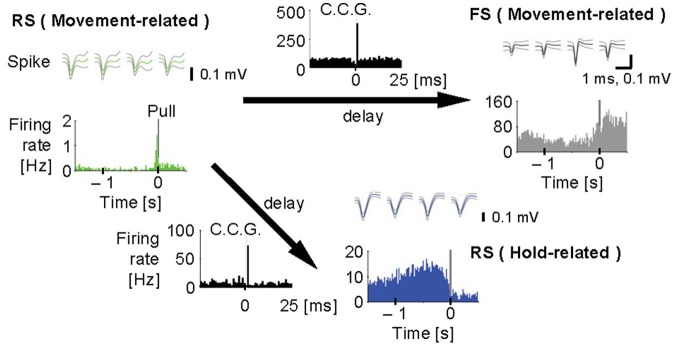
**Synchronous spiking among motor cortex neurons.** Cross-correlograms (black, C.C.G.; 1 ms bins) among a regular-spiking (RS) neuron with movement-related activity (green), an FS neuron with movement-related activity (gray), and another RS neuron with hold-related activity (blue). **Top**, spike waveforms [scale bars, 1 ms (the same for all spike traces) and 0.1 mV]. **Bottom**, pull-aligned (0 s) functional spike activity histogram (colored; 20 ms bins) in each neuron. Vertical bars indicate the onset of lever-pull. Each arrow means the direction of delay suggested by the peak in C.C.G. Y-axes indicate firing rate (Hz) in all the histograms. [Modified from Isomura et al. (2009)].

Furthermore, we analyzed multiunit activity in the motor cortex to examine functional interactions among putative excitatory and inhibitory cortical neurons during voluntary movements (Isomura et al., 2009). In the multiunit analysis, spikes were automatically isolated and clustered by our spike-sorting software (Takekawa et al., 2010, 2012), and then we classified the spike clusters into regular-spiking (RS) and FS neurons by the difference in spike width. The RS neurons in the neocortex appear to be mainly excitatory pyramidal cells, but also likely include inhibitory non-FS interneurons. Conversely, some pyramidal cells were recently shown to discharge thin spikes and may be misclassified as interneurons (Suter et al., 2012). Figure 4 shows that an example pair of one RS neuron and one FS neuron with movement-related activity increased the probability of their synchronous spiking within several milliseconds, which is represented by a single short-latency peak in their cross-correlogram. The RS neuron displayed similar synchronous spiking with another RS neuron with hold-related activity, too. Thus, motor cortex neurons discharged synchronously with functionally similar or different neurons, which happened in about 2% of possible neuron pairs. Such synchronous spiking between two neurons can be driven directly by monosynaptic excitation (Barthó et al., 2004) or indirectly by common excitatory inputs from the third neuron. In both cases, the highly effective synaptic excitation may be clarified by exceptional large-amplitude synaptic transmissions (Lefort et al., 2009; Morishima et al., 2011).

## Irregular neuronal firing in motor cortex

In the former section, we showed the dynamical behavior of neuronal networks in the primary motor cortex of rats performing a simple lever movement behavior. The results of *in vivo* electrophysiological recordings demonstrated that motor cortical neurons exhibit diverse functional neuronal subtypes in different layers and each neuronal subtype is activated concurrently, rather than sequentially, in the multiple layers. In this section, we turn to yet another question about the implications of irregular firing of a single motor cortex neuron for information coding. Namely, we propose that firing of cortical neurons distribute their firing rates to maximize the amount of total information represented by the distributed firing rates under some constraints, which we may call “the CMFE” hypothesis.

**Figure 5:**
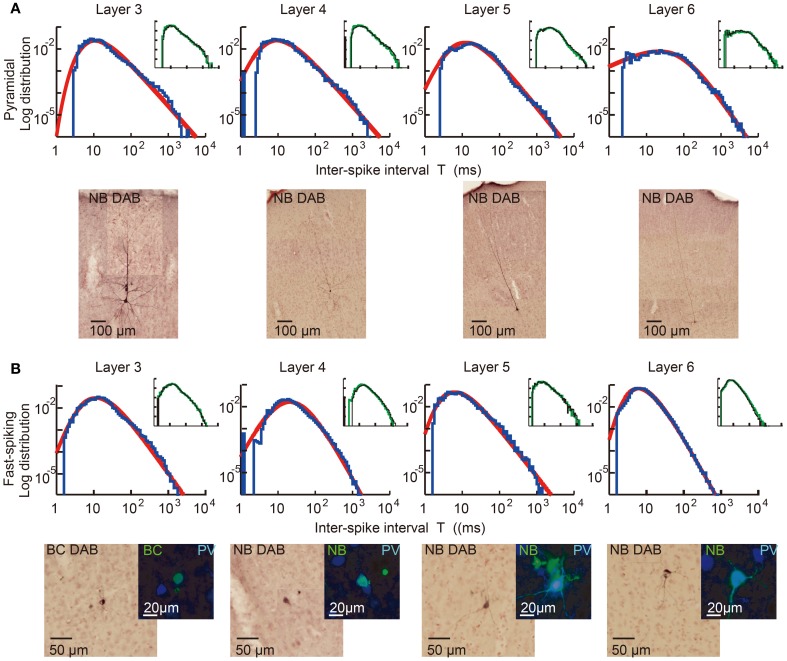
**Power-law ISI distributions of *in vivo* motor cortex neurons in different layers.** Juxta-cellular visualization and double-logarithmic plots of the ISI histograms (blue curves) of pyramidal neurons **(A)** and fast-spiking interneurons **(B)** recorded in layers 3, 4, 5, and 6 of rat motor cortex. The plots were fitted by neuron-dependent beta-2 distributions (red), and light micrographs display the morphological reconstruction of the eight neurons obtained by DAB (3,3′-diaminodbenzidine)-Nickel staining. The four neurons in **(B)** expressed parvalbumin (PV), a fast-spiking interneuron specific marker (blue: PV, green: biocytin or Neurobiotin). Inset of each panel represents the ISI distributions constructed from the 1st (black) and 2nd (green) halves of the same spike train. They prove the stationarity of the ISI distributions during the recordings. [Modified from Tsubo et al. (2012)].

We first explain key observations to the CMFE about the statistical property of irregular spike sequences in the motor cortex of behaving rats. For given statistics of input spike trains, distributions of inter-spike intervals (ISIs) represent a basic property of the responses of neurons. Poisson distributions or gamma distributions are generally considered to well represent the ISI distributions of cortical neurons. However, we recently found that the ISI distributions of the majority of motor cortex neurons show a power-law decaying long tail in behaving rats (Figure 5) (Tsubo et al., 2012). The power law of ISI distributions was found in more than half of the motor cortex neurons we recorded irrespective of their laminar locations and functional subtypes. Thus, a question was raised about whether the power-law ISI distributions have any implication for the information representation of cortical neurons. What message should we read out about cortical computation from such ISI distributions?

To answer this question, we employ the following double stochastic gamma (DSG) model and describe the ISI distributions of *in vivo* motor cortex neurons as:

(1) $$P_{\text{vivo}}(T)=\int_{0}^{\infty }q(T|r)P_{\text{vivo}}(r)dr,$$

where *T* stands for ISI. This equation regards a neuron as a translator of its internal state into an irregular output spike train of firing rate *r*, when irregular input spike trains set the neuron in the specific internal state. Note that here the firing rate *r* is regarded as a parameter that specifies neuron's internal state. For the consistency of the framework, the rate parameter should coincide with the firing rate of the neuron for stationary synaptic input. However, the value of *r* may vary dynamically and fluctuate in time for non-stationary synaptic input. Then, *P*_vivo_(*r*) is regarded as the probability that input to the neuron sets the value of the rate parameter equal to *r*, and *q*(*T*|*r*) refers to the ISI distribution at given rate *r* and represents an intrinsic property of the neuron in spike generation.

We may determine the expression of *q*(*T*|*r*) by measuring spike sequences of pharmacologically isolated neurons responding to a fluctuating input current that mimics balanced excitatory and inhibitory synaptic input. We indeed performed such recordings from a slice preparation of motor cortex and found that *q*(*T*|*r*) is given as a gamma distribution for the mean rate *r* (Miura et al., 2007). Then, Equation 1 implies that the ISI distribution of an *in vivo* cortical neuron is given as the convolution of the ISI distribution of *in vitro* neurons and the distribution of the instantaneous values of the rate parameter. This is a good approximation if the rate parameter changes its value only slowly compared to the cell's instantaneous rate of spike generation. If we use the fact that *P*_vivo_(*T*) is well expressed by a class of power-law distributions called “generalized beta-2 distribution”:

(2) $$P_{\text{vivo}}(T)\sim P_{\beta \text{2}}\left(T\right)=\frac{\tau^{\alpha }\Gamma (\alpha +\kappa )}{\Gamma (\alpha )\Gamma (\kappa )}\frac{T^{\kappa -1}}{{\left(T+\tau \right)}^{\alpha +\kappa }},$$

we can analytically show that *P*_vivo_(*r*) is well described as the gamma distribution with the mean *R* = α/κτ: *P*_vivo_(*r*) = [(α/*R*)^α^/Γ(α)]*r*^α − 1^ *e*^−α*r*/*R*^.

Now, an intriguing question arises. Why does *P*_vivo_(*r*) assume a form of the gamma distribution in the majority of motor cortex neurons? Does it happen to be so or is there any profound reason for that? In the next section, we show some evidence supporting for the latter.

## CMFE: a variational principle

Fundamental principles in physics and biology are often written in terms of variational principles in mathematics. For instance, in statistical physics the equilibrium state of a stochastic system is represented as a solution that minimizes the free energy, which is a function of the temperature and the microscopic variables describing the dynamical system. This view was also argued for the nonlinear dynamics of neural networks (Friston, 2010). In the present case, we consider the problem of minimizing the following objective function:

(3) $$J=-H[R]+\lambda_{r}\left(\bar{R}-R_{\max}\right)+\lambda_{e}\left(H[T|R]-H_{\max}\right)+\lambda_{0}\left(\int_{0}^{\infty }P_{s}(r)dr-1\right).$$

Below, we explain the meaning of each term in the right-hand side of the above functional *J*.

The first term in Equation 3 expresses the negative entropy of the time-varying firing rate of the neuron as a functional of the stationary distribution of firing rate *P*_*s*_(*r*):

(4) $$H[R]=-\int_{0}^{\infty }P_{s}(r)\ln P_{s}(r)dr.$$

We note that minimizing *J* essentially implies the maximization of the firing-rate entropy *H*(*R*). If this entropy is larger, the neuron is considered to use a wider variety of firing rates in its output spike train. The second term involves the average firing rate of the neuron

(5) $$\bar{R}=\int_{0}^{\infty }rP_{s}(r)dr,$$

and represents a constraint on the maximum value of the average firing rate. Since neuronal firing will require more energy at a higher frequency, this term imposes a limitation on the energy consumption of cell firing. The third term involves the conditional response entropy:

(6) $$H\left[T|R\right]=-\int_{0}^{\infty }P_{s}\left(r\right)\int_{0}^{\infty }q\left(T|r\right)\log q\left(T|r\right)dTdr,$$

which represents the average uncertainty of sequence of the ISIs generated by the neuron. The above quantity was also called “neuronal noise” (Borst and Theunissen, 1999). In this view, the function *q*(*T*|*r*) describes the degree of irregularity in the spike generation of the neuron at given firing rate *r*. Thus, the smaller the conditional response entropy is, the more reliable the spike generation is (Stevens and Zador, 1998). The last term in Equation 3 imposes the normalization condition on the probability density function *P*_*s*_(*r*).

To obtain the stationary firing-rate distribution *P*_*s*_(*r*) in CMFE, we have to find out such a *P*_*s*_(*r*) that minimizes *J* with Lagrange multipliers satisfying −∞ < λ_0_ < ∞ and λ_*r*_, λ_*e*_ ≥ 0. This minimization problem implies the maximization of the entropy of the firing-rate distribution *H*[*R*] under the constraints on the energy consumption (the maximum of average firing rate) and the maximum conditional entropy *H*[*T*|*R*]. For a scale-invariant ISI distribution q(*T*|*r*)d*T* = *f*(*Tr*)*r*d*T*, we can analytically solve the above maximization problem to find *P*_*s*_(*r*). Especially for the gamma ISI distribution *q*(*T*|*r*) = [(κ*r*)^κ^/Γ(κ)]*T*^κ − 1^ *e*^−κ*rT*^ (Miura et al., 2007), *P*_*s*_(*r*) is also given as a gamma distribution (Kuhn and Tucker, 1951): *P*_*s*_(*r*) ∝ *r*^λ_*e*_^ *e*^−λ_*r*_*r*^. Thus, *P*_*s*_(*r*) coincides with *P*_vivo_(*r*) after appropriate redefinition of the parameters.

## Implications of CMFE for neural information transmissions

The hypothesis of CMFE describes a solution to solve the cost-information trade-off in irregular neuronal firing. The CMFE hypothesis is an extension of the “maximum entropy of firing rate” with additional constraints on the conditional response entropy, where the firing-rate entropy means the variety of firing rates available for neuronal communication. The average energy consumed by a neuron may increase proportionally with firing rate. However, the conditional entropy will be increased for output spike trains if neurons use lower firing rate more frequently. Thus, our results imply that the firing rate values of motor cortex neurons are distributed so as to balance the tradeoff between the average uncertainty of output spike sequences and the energy consumption in spike generation.

The CMFE hypothesis is different from the so-called mutual information maximization (MIM). Mutual information between two probability variables represents the amount of information obtained for a variable by measuring the other. A widely adopted hypothesis is that neurons maximize mutual information between input and output (MacKay and McCulloch, 1952; Stein, 1967; Linsker, 1986; Bell and Sejnowski, 1995). According to MIM, noisy spiking neurons can maximize mutual information at given average firing rate only when it takes discrete values (Chan et al., 2005; Ikeda and Manton, 2009). However, such a discrete representation with firing rate does not seem to be consistent with our observations in rat motor cortex, and the CMFE hypothesis better accounts for the spike statistics of *in vivo* neurons in all layers of motor cortex.

## Discussion

We reviewed the PRCs of RS pyramidal neurons in layers 2/3 and 5 recorded from slice preparations of the rat motor cortex. The PRC is of particular interest since it gives some information on whether neurons may be synchronized or desynchronized with given synaptic input. We have found that the intrinsic response property of pyramidal neurons in oscillatory synchronization depends on the range of firing rates and the cortical layers they belong to. Interesting differences between cortical layers are observed in the PRCs in gamma band. Namely, in the frequency range from 20 to 80 Hz, layer 2/3 or layer 5 pyramidal neurons tend to possess type-2 or type-1 PRCs, respectively. These results imply that recurrent AMPA synaptic connections possibly promote synchronous neuronal firing of layer 2/3 pyramidal neurons in gamma band, but not that of layer 5 pyramidal neurons. The PRC type was also different in the theta frequency range. However, numerical simulations of a network model showed that the stable phase difference is close to 0 at low frequencies of 8–13 Hz even if the PRC belongs to type 1 (Tsubo et al., 2007a). Therefore, the layer-dependence of the PRC type may play an active role in synchronous firing at gamma frequencies, but not in other frequency ranges in rat motor cortex.

We may speculate possible implications of the above findings on PRCs for layer-specific cortical computations. The major portion of excitatory synapses on layer 2/3 pyramidal neurons originates from the surrounding layer 2/3 pyramidal neurons (Thomson and Bannister, 2003; Binzegger et al., 2004). Then, the layer 2/3 pyramidal neurons might serve as “resonant oscillators” (Izhikevich, 2000, 2004) through the synergistic effects of the rich recurrent synapses and the type-2 PRC. By contrast, layer 5 pyramidal neurons might operate as “integrators” since the type-1 neuron exhibits a continuous spectrum of firing rate from very low to high frequencies (Hodgkin, 1948; Tateno et al., 2004). These views seem to be consistent with the significant differences in firing rate between the superficial and deep layers of motor cortex in behaving rats.

Our observations in the microcircuit of motor cortex in behaving rats undoubtedly excludes the “layer-by-layer activation” hypothesis in which each layer of motor cortex has its own function for the movement phases, e.g., layers 2/3 for motor preparation and layer 5 for motor execution; instead, it supports “multi-layer activation” hypothesis that all the cortical layers cooperatively participate in every phase of the voluntary movement. Nevertheless, it is quite likely that a superficial layer circuit and a deep layer circuit may process the same information by different circuitry algorithms or for different functional purposes, since the spiking activity of deep layer neurons is generally much higher than that of superficial layer neurons in the neocortex. In fact, our preliminary data suggest that hold-related activity neurons are not distributed uniformly along the motor cortex layers (Igarashi et al., unpublished observation). Besides layer differences, it remains elusive whether the motor information simply flows in a one-way direction from superficial to deeper layers intracortically or reverberates for signal amplification or development through cortico-cortical and cortico-subcortical loops [cf., Weiler et al. (2008) and Anderson et al. (2010) for layer-specific connectivity in motor cortex]. In addition, we recently found that most motor cortex neurons were phase-locked to gamma oscillations of LFP (Igarashi et al., unpublished observation). It suggests that these neurons may communicate in a microcircuit through spike synchronization on a specific phase of the gamma oscillations. As neocortical gamma oscillations are widely influenced by the hippocampal theta activity (Sirota et al., 2008), synchronous spiking during gamma oscillations may be a common mechanism underlying neuronal communications in the neocortex and hippocampus.

Finally, we have proposed CMFE to account for the power-law statistics of irregular firing of motor cortex neurons. The hypothesis of CMFE yields a novel view of the way neurons translate information on firing rate into irregular spike trains. A significant advantage of the brain over modern supercomputers is the very low power consumption. While the storage and transmission of information should be very accurate in electric computers, the CMFE does not require a very high accuracy. Artificial information machines require the precision at the expense of the amount of representable information, i.e., the entropy, whereas the brain likely possesses a variety of communication windows at the expense of the transfer information. The CMFE suggests a mathematical principle for neural information coding alternative to the maximization of mutual information.

Our results explained in this article have revealed some key features of the layer-specific information processing in the microcircuit of motor cortex. However, the basic circuit design for this information processing still remains largely unknown. In particular, we predict a link between the CMFE hypothesis for irregular spiking and the multi-band oscillations observed in motor cortex. In this respect, it is intriguing to examine whether the CMFE hypothesis is valid for sensory cortices, which often display significant gamma oscillations. What aspect of motor information is processed in each layer? How does such information flow between different layers? How does each layer of motor cortex communicate with other cortical and subcortical regions? All these questions should be answered more clearly in future studies.

### Conflict of interest statement

The authors declare that the research was conducted in the absence of any commercial or financial relationships that could be construed as a potential conflict of interest.
